# The Black identity, hair product use, and breast cancer scale

**DOI:** 10.1371/journal.pone.0225305

**Published:** 2019-12-04

**Authors:** Dede Teteh, Marissa Ericson, Sabine Monice, Lenna Dawkins-Moultin, Nasim Bahadorani, Phyllis Clark, Eudora Mitchell, Lindsey S. Treviño, Adana Llanos, Rick Kittles, Susanne Montgomery

**Affiliations:** 1 Department of Population Sciences, Division of Health Equities, City of Hope Comprehensive Cancer Center, Duarte, California, United States of America; 2 Department of Psychology, University of Southern California, Los Angeles, California, United States of America; 3 School of Behavioral Health, Loma Linda University, Loma Linda, California, United States of America; 4 Department of Health Sciences, California State University-Northridge, Northridge, California, United States of America; 5 Healthy Heritage Movement, Riverside, California, United States of America; 6 Quinn Community Outreach Corporation, Moreno Valley, California, United States of America; 7 Rutgers School of Public Health and Cancer Institute of New Jersey, Piscataway, New Jersey, United States of America; University of California Los Angeles, UNITED STATES

## Abstract

**Introduction:**

Across the African Diaspora, hair is synonymous with identity. As such, Black women use a variety of hair products, which often contain more endocrine-disrupting chemicals than products used by women of other races. An emerging body of research is linking chemicals in hair products to breast cancer, but there is no validated instrument that measures constructs related to hair, identity, and breast health. The objective of this study was to develop and validate the Black Identity, Hair Product Use, and Breast Cancer Scale (BHBS) in a diverse sample of Black women to measure the social and cultural constructs associated with Black women’s hair product use and perceived breast cancer risk.

**Methods:**

Participants completed a 27-item scale that queried perceptions of identity, hair products, and breast cancer risk. Principal Component Analyses (PCA) were conducted to establish the underlying component structures, and confirmatory factor analysis (CFA) was used to determine model fit.

**Results:**

Participants (n = 185) were African American (73%), African, and Caribbean Black women (27%) aged 29 to 64. PCA yielded two components that accounted for 61% of total variance. Five items measuring *sociocultural perspectives about hair and identity* loaded on subscale 1 and accounted for 32% of total variance (α = 0.82; 95% CI = 0.77–0.86). Six items assessing *perceived breast cancer risk related to hair product use* loaded on subscale 2 and accounted for 29% of total variance (α = 0.82 (95% CI = 0.74–0.86). CFA confirmed the two-component structure (Root Mean Square Error of Approximation = 0.03; Comparative Fit Index = 0.91; Tucker Lewis Index = 0.88).

**Conclusions:**

The BHBS is a valid measure of social and cultural constructs associated with Black women’s hair product use and perceived breast cancer risk. This scale is useful for studies that assess cultural norms in the context of breast cancer risk for Black women.

## Introduction

Breast cancer remains the second leading cause of death in the United States among women [1, 2], with Black women being particularly vulnerable [3]. Recent reports indicate that while the incidence rates of breast cancer in Black and White women have converged [4], mortality rates among Black women are at least 40% higher than their White counterparts [3]. Furthermore, Black women are more likely to be diagnosed with and die from more aggressive forms of breast cancer than White women who are more often diagnosed at earlier stages [5]. Research on cancer disparities has not been able to conclusively explain Black women’s elevated risk of breast cancer mortality or their more aggressive phenotypes, but differences in prognosis and etiology have been correlated with race [6], genetics [7], lifestyle and behavioral factors [8], and environmental exposures [9].

One environmental factor that has been gaining increasing attention in breast cancer research is hair and personal care products [9–11]. A growing body of literature [9–14] supports an association between use of some hair care products (e.g., hair dyes, relaxers, and deep conditioners) and breast cancer risk. Data from animal models [15–17] suggest exposures to compounds found in some hair products, particularly those containing endocrine-disrupting chemicals (EDCs) and chemicals with mutagenic properties, may be important etiologic risk factors for several human cancers, including breast cancer. Stiel and colleagues’ review of hair product use and breast cancer risk included research on environmental estrogen, EDCs, and placenta-derived ingredients found in hair products used primarily by African American women. The authors concluded there is significant evidence to support the role of hair product use in the risk of early-onset breast cancer in Black women.

In addition, Myers et al. [14] assessed ethanol extracts of eight personal care products frequently used by African Americans for estrogenic and anti-estrogenic activity in a human breast cancer cell line. They detected estrogenic activity in oil, hair lotion, extra-dry skin lotion, intensive skin lotion, and petroleum jelly, and anti-estrogenic activity in placenta hair conditioner, tea-tree hair conditioner, and cocoa butter skin cream. The authors concluded some hair and skin care products have ingredients that can mimic estrogen functioning. Llanos and colleagues [11] also examined the association between breast cancer risk and use of hair dye, chemical relaxers, and deep conditioners in a sample of African American and White women. They found, among the dark hair dye shades, African American use was associated with increased breast cancer risk (OR = 1.51, 95% CI: 1.20–1.90).

Despite the mounting evidence of possible health risk, hair products remain popular in the Black community [10, 11, 18]. Compared to white women, Black women invest more money on hair products [19], are more likely to use relaxers and deep conditioners, and use them at younger ages [11, 18]. As a result, Black women are more likely to be exposed to hormonally active chemicals in hair products across their lifespan, potentially increasing their risk for developing breast cancer [18].

The literature on Black women’s attitude to their hair offers mixed explanations for the popularity of hair care products in Black communities. Some scholars suggest hair across the African diaspora is synonymous with identity, individuality, and beauty norms [20, 21]. For many Black women, maintaining hair with sculpting and straightening products is an extension of self and a way to achieve social acceptance [21, 22]. Alternatively, scholars such as Johnson and Bankhead challenge the notion that straight hair represents an ideal form of beauty and is connected to social acceptance [22]. They argue that wearing hair in its natural state is the “new normal” that celebrates Black identity and pride. But even products designed for natural Black hair have been found to contain potentially harmful ingredients [23]. So, whether Black women choose to chemically alter their hair or keep it in its natural state, they are overexposed to hair products containing endocrine disrupting and other toxic chemicals—many of which are not listed on product labels as they are usually listed generically as fragrances [10, 24].

There is a gap in our understanding of how these environmental, sociocultural, and biological factors converge and impact breast cancer risk and outcomes among Black women. Currently, there is no validated scale that measures constructs related to hair, identity, and breast health. Instruments have been developed to measure related but general issues, such as social and personal identity [25], health beliefs [26], and perceptions of health risks [27]. However, none of these instruments capture the constructs that emerge at the intersection of social, cultural, and health-related factors. Having a validated tool that measure these latent structures will provide a more comprehensive understanding of Black women’s breast cancer risk. The purpose of this study was to validate the Black Identity, Hair Product Use, and Breast Cancer Scale (BHBS) in a diverse sample of Black women to provide a measure that captures the sociocultural factors related to perceived breast cancer risk and hair product use.

## Materials and methods

This study was part of a broader project that used a mixed method design and community-based participatory research (CBPR) principles to evaluate the association between hair products use and perceived breast cancer risk among Black women in Southern California. The study was conducted in two phases: a qualitative phase followed by a quantitative phase. Findings from the qualitative phase were used to inform quantitative data collection. This design is particularly useful for developing a new scale [28]. The project was conducted by three co-investigators one from an academic institution and two from African American community organizations. The original research questions were developed by community stakeholders who were concerned about the growing number of breast cancer diagnoses in their region.

To ensure all research activities were within ethical guidelines, an application detailing the project was submitted and approved by the Loma University Institutional Review Board (IRB). Every participant provided written informed consent. All individuals involved with data collection were certified (staff and community research navigators, aka navigators) in research ethics using an IRB approved curriculum developed specifically for CBPR data collection [29].

### Participants recruitment and procedures

Recruitment for the broader study took place in two stages. In stage 1, we used purposive sampling to recruit participants (n = 125) who self-identified as African American, African, or Afro-Caribbean. In stage 2, we used snowball and convenience sampling to recruit African American, African, or Afro-Caribbean men (n = 66) and women (n = 211). The social networks of the two community co-principal investigators agencies were used to recruit participants. For both stages, participants were primarily recruited face-to-face at churches, hair salons, community meetings, women’s conferences, hair and education showcases, and other areas where the target population congregated. Also, flyers were posted in these settings and emailed to listservs of the community investigators. For stage 1, participants received $20 in the form of store cards (e.g. Target; Food 4 Less). Participants were not compensated for their participation in stage 2 of the study. For both stages, data were collected in churches, beauty salons, and community meetings.

The analysis described in this paper includes only Black women with or without a history of breast cancer (n = 185). Women were excluded from the study if they did not self-identify as African American, African, or Afro-Caribbean.

### Source of data and scale measures

In Phase 1 of the study, we explored the cultural and personal meaning of hair in the Black community. Interview questions were developed by participant type (i.e., women with and without a history of breast cancer and their male partners, hair stylists, and salon owners) [30]. Questions were piloted, and modified based on participants and community navigator feedback. Interviews were audiotaped, and field notes drafted after focus group and key informant interviews.

Navigators were trained in basic qualitative and quantitative research approaches including the development of semi-structured interview guides, interviewing skills, the codebook development for qualitative data analysis, and research team trainings on how to use QDA-miner—a qualitative data analysis software [31] that was used for data analyses. Navigators held credentials ranging from high school to master’s level education. At the time of the study, navigators were part-time employees of the two community organizations of the project. Some navigators were retired, while others were full-time doctoral students at local universities. Navigators were also predominantly female, but one male navigator completed the interviews with male participants. Only participants, navigators, and co-principal investigators were present during data collection.

To achieve triangulation, the navigators used a common semi-structured interview guide[32] adjusted to the target audience to conduct key informant interviews and focus groups. Target groups included women with and without a history of breast cancer, their male partners, hair stylists, and salon owners. While the interviews explored perceptions of the causes of breast cancer, participants’ relationship with hair and hair product use, and perceptions of the potential harmfulness of hair care products, the focus groups were used for validation and member checking. Navigators were not blinded to participants’ breast cancer diagnoses. Data were collected in 2013 through early 2014.

Interviews and focus groups were audio recorded and transcribed verbatim to maintain accuracy. We used QDA-Miner to code and analyze the transcripts using a Grounded Theory approach [33]. Following open coding, preliminary results were presented to the navigators, discussed with the research team, and organized into related concepts or themes that were used to inform the quantitative instrument development. The study team engaged in thoughtful deliberations to resolve theme disagreements. Transcripts were not returned to participants for comments and corrections although participant feedback was obtained during the focus groups. The most prominent themes were the critical role of hair for Black women; the relevance of the project to the community; and the notion that “everything causes cancer”, so why change [20].

After the themes from the qualitative results were identified, the investigator from the academic institution guided the community navigators in a series of discussions that resulted in the development of questionnaire items. Items were grouped into two categories:1) perceived risk, knowledge, and attitudes about Black hair and breast cancer (8 items); and 2) perceptions about hair and Black culture (19 items). Questions pertaining to perceived risk, knowledge, and attitudes about Black hair and breast cancer included items such as “All Black women should worry about the ingredients in hair products”; and “Because of breast cancer, I intend to watch the ingredients of the products I will use”. Questions pertaining to perceptions about hair and Black culture explored beliefs about Black hair in the workplace, hair product use, and cultural and personal attitudes related to hairstyles. Items included “Hair has a special role in Black culture”; “My hair is a cultural reflection of who I am”; and “I do not care how much I spend on hair products.” For all questions, the Likert scale response options were *Strongly disagree*, *Disagree*, *Agree*, and *Strongly agree*. The 27-item scale discussed here was one component of a larger 42-item survey instrument. Other questions explored respondent’s lifestyle, health behaviors, BC knowledge, demographics, and family medical history. Questions were reviewed for clarity among the research team and pilot tested prior to their use in the final survey.

In Phase 2, navigators used the 42-item questionnaire developed in Phase 1 to collect data in settings previously mentioned. Each questionnaire took approximately 30–45 minutes to complete and the majority (approximately 98%) were completed in person. Those that were not completed in person were submitted electronically or via postal mail. Data were collected from 2015 through 2016.

### Demographic measures

Education level was assessed using the question: “What is the highest level of education you have completed?” Response options were: *Some high school*, *High school diploma (or equivalent)*, *Some college*, *College degree*, *Graduate degree*, and *Professional certification (cosmetology*, *etc*.*)*. Age was denoted in *years*. Household income question was: “Please check the range of income that is closest to your own:” with the following response options: *Less than $25*,*000*; *$26*,*000-$50*,*000*; *$51*,*000-$75*,*000*; *$76*,*000-$100*,*000*; *$100*,*000-$150*,*000* and *More than $151*,*000*. Insurance status was assessed using the question: “Do you have health insurance?” With response options *Yes* or *No*. Birth place was determined using the question: “Were you born in the United States?” With response options *Yes* or *No*. The question, “Have you been diagnosed with cancer?” With response options *Yes* or *No*, was used to assess diagnosis status. Family history of breast cancer was determined using the question: “Have any of your family members been diagnosed with breast cancer?” With response options *Yes* or *No*. The question “Have you ever had a mammogram?” was used to assess participant breast cancer screening history with response options *Yes* or *No*. Product use information was obtained using the question “How often do you use the following products?” Product types included wash out conditioner, leave in conditioner, relaxer from salon, do it yourself relaxer, detangler, damaged hair treatment (i.e. hair mayonnaise), do it yourself hair dye kits, and professional hair coloring. Response options included 1) *Several times a week*, 2) *Daily*, 3) *Several times a month*, 4) *Several times a year*, 5) *Used to/stopped using* and 6) *Never used it*. Response options 1–5 were recategorized as *Yes* for product use and response option 6 was recategorized as *No* for product use in analyses.

### Principal components analysis

In order to examine the underlying structure and dimensions of identity, hair products use, and perceived breast cancer risk, a Principal Component Analysis (PCA) was conducted using SPSS 24.0 [34]. As this is the first analysis of its kind to examine the cultural influence of hair product use among Black women, several model-fitting techniques were tested, including both orthogonal and oblique rotations. An oblique rotation (e.g. Promax) allows components to be intercorrelated, while an orthogonal rotation (e.g. Varimax) minimizes the number of variables with high loadings and simplifies the solution [35]. As a significant dearth of similar validation studies exists in the literature—and thus no factor analytic studies with which to compare—we examined several exploratory models during this first analysis phase, including both Promax and Varimax rotations.

Several criteria were used to examine the number and combination of items in each component, including Horn’s parallel analysis[36], a scree plot of Eigenvalues[37], Kaiser criterion[38], and factor loadings[39]. The criterion cutoff was set at ±0.35 for the item loadings. Based on this criterion, each item loaded most highly on one of two distinct components, with many items loading poorly (<±0.20) on additional components. The poorly loaded items were dropped and models were re-assessed each time by calculating inter-item reliability of the items for each component based on Cronbach’s alpha (α), which indexes internal scale consistency or the extent to which a scale measures an underlying construct [40, 41].

## Results

Confirmatory factor analyses (CFA) were performed using Mplus software [42] to determine the factor solution that best explains the Black Identity, Hair Product Use, and Breast Cancer Scale. Based on the initial PCA results, including the criteria as outlined above, two and three-factor solutions were examined and compared to one another. Goodness of fit was determined by several indices: chi-square (χ2), Akaike’s Information Criterion (AIC), Root Mean Square Error of Approximation (RMSEA) of <0.10, Comparative Fit Index CFI of >0.90, and Tucker Lewis Index (TLI) of >.90 [43–45]. The χ^2^ assess overall fit and the discrepancy between the sample and fitted covariance matrices. In addition to the *χ*^2^ test, the AIC is compared for each fitted model[46]. The AIC is a widely used index of fit, such that smaller AIC values are indicative of the most parsimonious and well-fitting model.

### Demographic

The final analytic sample (n = 185) was comprised of 73% African American and 27% Caribbean and African Black women aged 29 to 64 years (Table 1). Participants were highly educated with over 50% of participants having a college or a more advanced degree. In addition, most participants reported use of hair products. Overall, participants reported using wash out conditioner (89.7%), leave in conditioner (83.8%), relaxer (70.8%), do it yourself relaxer (58.4%), detangler (63.2%), treatment for damaged hair(64.3), do it yourself hair dye kits(50.8%), and professional hair coloring(55.1%).

**Table 1:** Demographic characteristics of participants (N = 185).

Demographics	African American n = 135 (73.0%)	Caribbean/African n = 50 (27.0%)	p-value
**Education level**			.556
≥ Some high school	9(6.7)	3(6.4)	
≤ College degree	73(54.1)	21(44.7)	
Graduate degree Professional certification	42(31.1) 11(8.1)	20(42.6) 3(6.4)	
**Age**			.326
29 and below	16(11.9)	7(14.0)	
30–39	28(20.7)	17(34.0)	
40–49 50–59	28(20.7) 25(18.5)	10(20.0) 6(12.0)	
≥60	38(28.0)	10(20.0)	
**Household income**			.526
Less than $25,000	30(23.1)	12(27.3)	
$26,000-$50,000	42(32.3)	9(20.5)	
$51,000-$75,000 >$75,000	28(21.5) 30(23.1)	11(25.0) 12(27.3)	
**Insurance status**			.516
Yes	127(96.2)	47(94.0)	
No	5(3.8)	3(6.0)	
**Birth place**			.001
US	121(91.7)	9(18.4)	
Non-US	11(8.3)	40(81.6)	
**Breast cancer screening**			.527
Yes	55(64.7)	19(57.6)	
No	30(35.3)	14(42.4%)	
**Breast cancer diagnosis**			.520
Yes	12(9.1)	3(6.1)	
No	120(90.9)	46(93.9)	
**Family history of breast cancer**			.001
Yes	58(45.3)	9(18.8)	
No	70(54.7)	39(81.3)	

Percentages and number of participants do not equal total due to missing data.

### PCA and CFA

**Fig 1:**
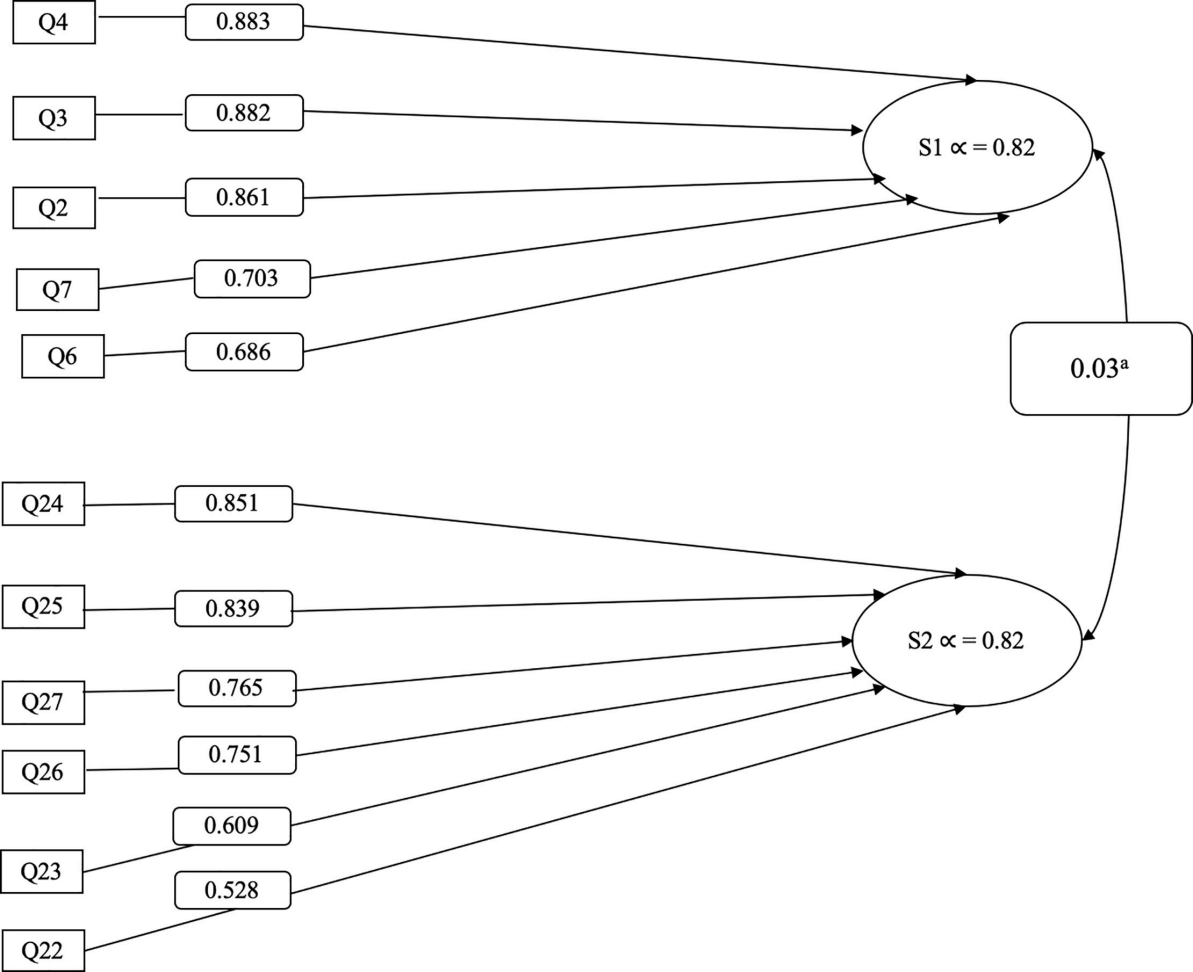
Diagram of the 11-item Black identity, hair product use and breast cancer scale. (S1, Subscale 1): Sociocultural perspective about hair and identity. (S2, Subscale 2): Perceived breast cancer risk related to hair product use. ^a^The interrelationship between S1 and S2.

We examined the factor structure of the 27 items with an aim to better understand the structure and dimensionality of identity, perceived risk, and attitude regarding Black hair and perceived breast cancer risk. According to fit criteria, PCA with Varimax rotation yielded two distinct components that accounted for 61% of the total variance, with 11 of the 27 items loading highly on these two unique components. A parallel analysis also indicated a two-component solution, though 1-component and 3-component solutions were also examined in subsequent CFA[36]. The remaining 16 items were dropped due to poor fit (<±0.35), which led to an improvement in internal scale consistency. Table 2 presents the means, standard deviations, skewness and kurtosis for the initial 27 items, Fig 1 presents factor loadings for the 11 items, and Table 3 presents loadings from PCA for two extracted subscales.

**Table 2:** Means, Standard Deviations (SD), Skewness, and Kurtosis for the initial 27-items of the Black identity, hair product use, and breast cancer scale.

		Mean (SD)	Skewness (Kurtosis)
Q1	Hair has a special role in Black culture.	3.43 (.792)	-1.59 (2.39)
**Q2**	**Black men do not like Black women to wear their hair natural.**	**2.11 (.853)**	.368 (-.354)
**Q3**	**In order to be successful in business, it is a necessary for Black women to have** **their hair straight.**	**2.05 (.878)**	**.547 (-.354)**
**Q4**	**In order for Black women to attract Black men, they need to straighten their hair.**	**1.94 (.782)**	**.704 (.403)**
Q5	If I had a daughter, I would want them to straighten their hair.	1.66 (.697)	.792 (.370)
**Q6**	**Black women feel pressure from their female friends to straighten their hair.**	**2.30 (.835)**	**.067 (-.623)**
**Q7**	**Black women feel pressure from their partners to straighten their hair.**	**2.20 (.825)**	**.255 (-.503)**
Q8	White people are intimidated when they see Black hair in its natural state.	2.46 (.871)	-.063 (-.677)
Q9	Keeping up with current hairstyles is important in Black culture.	2.78 (.884)	-.438 (-.433)
Q10	My hair is a cultural reflection of who I am.	2.86 (.857)	-.530 (-.203)
Q11	Older members of my family would disapprove if I wore my hair natural.	1.98 (.926)	.714 (-.292)
Q12	I believe natural hair can be professional as well as straightened hair.	3.41 (.795)	-1.37(1.48)
Q13	Black women should not be concerned about their hair in professional settings.	2.46 (.960)	.226 (-.902)
Q14	In the work setting, I get treated differently based on how my hair is styled.	2.34 (.929)	.268 (-.747)
Q15	I do not care how much I spend on hair products.	2.11 (.914)	.490 (-.542)
Q16	I do not think chemically altered (relaxer, perm, texturizer, color) hair is harmful.	1.78 (.875)	.989 (.292)
Q17	I am completely satisfied with my current hairstyle.	3.09 (.799)	-.661 (.080)
Q18	I believe natural hair is healthier than chemically altered.	3.42 (.761)	-1.28 (1.29)
Q19	Black women spend too much money on hair products.	2.86 (.972)	-.434 (-.809)
Q20	I believe there is enough information about breast cancer risk and hair products for Black women.	1.90 (.880)	.893 (-1.10)
Q21	I would be interested in education regarding the potential risk between hair products and breast cancer.	3.31 (.748)	-1.10 (1.30)
**Q22**	**I am concerned that the labels of hair care products do NOT list all the ingredients.**	**3.08 (.838)**	**-.989 (.815)**
**Q23**	**Because I am concerned about breast cancer, I plan to go natural (style my hair without chemicals).**	**2.88 (.886)**	**-.369 (-.625)**
**Q24**	**Because I am concerned about breast cancer, I intend to watch the ingredients of the products I will use.**	**3.25 (.742)**	**-.801 (.468)**
**Q25**	**All Black women should worry about the ingredients in hair products.**	**3.35 (.664)**	**-.900 (1.22)**
**Q26**	**Because I am concerned about breast cancer, I plan to adjust how I use hair care products.**	**3.20 (.709)**	**-.705 (.648)**
**Q27**	**I want to learn more about the risk hair products can cause to my health.**	**3.44 (.633)**	**-1.10 (2.05)**

Note: 11-item Black identity, hair product use and breast cancer scale questions are bolded.

Subscale 1: Sociocultural perspective about hair and identity [Questions: 2, 3, 4, 6, 7].

Subscale 2: Perceived breast cancer risk related to hair product use [Questions: 22–27].

**Table 3:** Loadings from PCA for two extracted subscales for the 11-item Black identity, hair product use, and breast cancer scale.

		S1	S2
Q2	Black men do not like Black women to wear their hair natural.	.861	-.245
Q3	In order to be successful in business, it is a necessary for Black women to have their hair straight.	.882	-.232
Q4	In order for Black women to attract Black men, they need to straighten their hair.	.883	.201
Q6	Black women feel pressure from their female friends to straighten their hair.	.686	.101
Q7	Black women feel pressure from their partners to straighten their hair.	.703	.121
Q22	I am concerned that the labels of hair care products do NOT list all the ingredients.	.222	.528
Q23	Because I am concerned about breast cancer, I plan to go natural (style my hair without chemicals).	.232	.609
Q24	Because I am concerned about breast cancer, I intend to watch the ingredients of the products I will use.	.343	.851
Q25	All Black women should worry about the ingredients in hair products.	.301	.839
Q26	Because I am concerned about breast cancer, I plan to adjust how I use hair care products.	.251	.751
Q27	I want to learn more about the risk hair products can cause to my health.	.345	.765

Subscale 1 (S1): Sociocultural perspective about hair and identity [Questions: 2, 3, 4, 6, 7].

Subscale 2 (S1): Perceived breast cancer risk related to hair product use [Questions: 22–27].

The first component, which consisted of five items representing sociocultural perspective about hair and identity, accounted for 32% of the total variance and demonstrated robust reliability (α = 0.82, CI = 0.77–0.86). The second component accounted for an additional 29% of the total variance and consisted of six items that measure perceived breast cancer risk related to hair product use. Cronbach’s alpha for the six-item subscale was 0.82 (CI = 0.74–0.86).

CFA confirmed a two-component model fit the data according to fit statistics for these 11 items (χ^2^ = 87.225, p-value = <01; AIC = 4219.615; RMSEA = 0.07; CFI = 0.91; TLI = 0.88). Both one-component (χ^2^ = 165.162, p-value < .01; AIC = 4295.552; RMSEA = 0.13; CFI = 0.75; TLI = 0.69) and three-component models (χ^2^ = 160.926, p-value = < .01; AIC = 4297.316; RMSEA = 0.13; CFI = 0.76; TLI = 0.66) were also tested but neither fit the data well according to the fit criteria. The interrelationship between the two confirmed subscales was 0.03 (not significant), demonstrating that each subscale represents a distinct component with no significant intercorrelation at the higher-order level. Given the patterns in both PCA and CFA, we concluded that two subscales were represent in these 11 questions: S1. *Sociocultural perspective about hair and identity*, and S2. *Perceived breast cancer risk related to hair product use*.

## Discussion

The Black Identity, Hair Product Use, and Breast Cancer Scale (BHBS) composed of 2 subscales and 11 items, was validated in this study using the methodology utilized in psychometry[35, 37, 40, 41]. To our knowledge, the BHBS is the first scale that evaluates the sociocultural factors related to perceived breast cancer risk and hair product use. Two distinct subscales were uncovered that measure the underlying components of the cultural influence of hair product use among Black women.

Subscale 1, *Sociocultural perspective about hair and identity*, consists of five items representing a sociocultural perspective regarding hair and identity with items like “Q3. In order to be successful in business, it is a necessary for Black women to have their hair straight” and “Q7. Black women feel pressure from their partners to straighten their hair.” This component reflects a distinct dimension that measures hair and identity constructs. Subscale 2, *Perceived breast cancer risk related to hair product use* is comprised of six items that assess perceptions of risk associated with hair products. Items that loaded highly on this distinct component included questions like “Q24 Because I am concerned about breast cancer, I intend to watch the ingredients of the products I will use” and “Q25 All Black women should worry about the ingredients in hair products”. These reflect a dimension that measures perceived risk and product usage specifically. Both dimensions were found to describe these data, with additional dimensions (e.g. 3-Subscale model) leading to degradation in fit. Importantly, the initial PCA model fitting also revealed that more than one component was required to describe the structure of these data. This specifically showed that two components are needed because each reflects a distinct construct that assesses different underlying mechanisms and perceptions.

Furthermore, the topic of hair in the Black community has always represented varying degrees of prominence, ranging from politically charged euphemism to exhortation of African pride [20–22]. Therefore, it comes as no surprise that Black women invest millions of dollars on products that craft their projection of identity to society [19]. According to cultural norms, Black women have to spend time on their hair [20]. This affects not only their personal life, but also how they are perceived professionally. Since hair carries such strong political and sociocultural meanings in the Black community [20, 23], research on hair products and breast cancer must also consider issues of identity and the trade-off between perceptions of risk and ideals of beauty. This newly developed Black Identity, Hair Product Use, and Breast Cancer Scale (BHBS) provides a validated instrument that can be used to help untangle these complexities.

Overall, this scale acknowledges that both perceived health risk associated with hair products and the sociocultural significance of hair are critical to Black women. Culture is often regarded as the defining element of an individual’s actions that reflects their beliefs, attitudes, and normative behavioral patterns [47]. This study adds to the hair-health literature by emphasizing the critical importance of recognizing beliefs and attitudes in research on breast cancer and hair product use in Black communities.

We hope that the BHBS will help increase our understanding of the interplay between identity, health, and behavioral intentions for Black women, since it provides targeted questions that measure women’s perceptions regarding breast health and identity. With the increasing prominence of research on Black hair and breast health it is critical to take these issues into account. It is also important to note that our women acknowledged both a socio cultural perspective as well as a breast cancer risk perspective in their responses. As the discussion about hair product risk for breast health matures we need to measure and then weigh both, as both clearly affect women’s perspective on the issue. Moreover, since perception precedes and influences behavior, this scale has utility for community-based interventions aimed at promoting healthier hair care alternatives. Through the parent study, for example, stylists and salon owners expressed interest in learning about less harmful products to use on their clients’ hair. This scale can capture data that may help researchers and community based-organizations gauge community members’ willingness to adopt safer products as the field is moving from exploration of risk for breast cancer to community based prevention education.

While awareness on the potential hazards of hair and personal care products is increasing, interventions geared towards minimizing risk and exposure should be developed in parallel. It was only 80 years ago (1940s/1950s) that studies from animal experiments, chemical analyses, and epidemiology concluded that cigarettes were the cause of lung cancer [48]. Despite the substantial evidence, only one-third of U.S. doctors up until the 1960s believed this conclusion, potentially due to propaganda by the tobacco industry to salvage cigarette sales. As a result, an estimated 6 trillion cigarettes smoked in 1990 caused about 6 million deaths in 2015 (one death/5 seconds). Comparably, with increasing evidence pointing to a risk connection between the use of hair products and risk for breast cancer in Black women, to wait until definitive evidence is provided on the health consequences of hair and personal care products before intervening, would be irresponsible and potentially detrimental to communities of color. In particular, as exposure to these hazardous chemicals is substantially higher in these communities than other groups and occurs as early as in utero.

There are several limitations that should be considered in the interpretation of findings from this study, including the relatively small group of participants. We also lack participation of a larger group of women with a history of breast cancer. It is recommended for future studies, that women with breast cancer represent a larger percentage of the sample. As many people experience hair loss when receiving treatment for breast cancer [32], a question for this group of Black women is how they continue to relate to a culture that places so much value on hair? Another limitation is inherent to a study of this nature in which a new scale is created, evaluated, and validated. While the methodological design follows a common data analytic path, there are no other studies with which to compare our results. Therefore, future studies that will use the scale are warranted to examine not only the structure and subscales further, but also examine the scale’s impact in explaining decision-making to reduce risk. In addition, it is important to acknowledge that our sample size lacked sufficient power to analyze the PCA and CFA with different samples. Though it is common in the literature overall[49], future studies should seek to have larger samples and further investigate the scale in a split sample. Furthermore, our study population lacked socioeconomic (e.g., employment status, education) diversity, our instrument did not include questions about breast cancer type and our focus on only Black women in Southern California limits the generalizability of our findings.

While the findings of this study are limited by the issues stated above, there is currently no scale examining the relationship between hair, identity and perceived breast cancer risk. As research in the hair-health discipline continues to expand, social/behavioral and basic scientists may benefit from use of this tool.

## Conclusion

Black women continue to be diagnosed and die from more aggressive forms of breast cancer than White women [5]. There is growing interest in more fully understanding how environmental exposure to chemicals in hair and personal care products is associated with breast health among Black women [9–11, 14]. This has resulted in a surge of social/behavioral and basic science research studies that are raising awareness about the potential risks of hair and other beauty products in the Black community. For instance, Myer’s et al. findings suggest off the shelf hair and skin care products can have estrogenic and anti-estrogenic properties [14]. Helm and colleagues’ recent study suggests the products used by Black women have higher levels of chemicals not reported on the labels. These chemicals are regulated by California’s Proposition 65 or prohibited by European Union cosmetic regulations [10], but minimally regulated by the Food and Drug Administration. The products have also been found to exhibit estrogenic properties that could increase the risk of cancer for the consumer. Similarly, Breast Cancer Prevention Partners recently reported personal care products—marketed to children of color and endorsed by celebrities or deemed “green” or good for the environment—actually contain more hazardous chemicals than the cleaning products they tested [24]. The concern about the potential risks of hair and personal care products extends beyond the academy [9–11] to communities of color, wherein Black men and women’s concerns initiated this collaborative study.

However, all behavior change is complicated and subject to many influences, even in light of emergent scientific insights. As we seek to better understand cultural influences that perpetuate breast cancer disparities, the impact of hair on identity for Black women should also be recognized. Hair is a fundamental component of one’s cultural identity. Our Black Identity, Hair Product Use, and Breast Cancer Scale may provide important insights into the social and behavioral patterns as well as the growing perceptions about risk of Black women in relation to breast cancer prevention and hair product risk. Considering both of these perspectives of women across the African diaspora may yield culturally appropriate strategies that inform interventions about identity, hair product use, and breast cancer risk.
